# Loss of NMDA receptors in dopamine neurons leads to the development of affective disorder-like symptoms in mice

**DOI:** 10.1038/srep37171

**Published:** 2016-11-17

**Authors:** Kamila Jastrzębska, Magdalena Walczak, Przemysław Eligiusz Cieślak, Łukasz Szumiec, Mateusz Turbasa, David Engblom, Tomasz Błasiak, Jan Rodriguez Parkitna

**Affiliations:** 1 Laboratory of Transgenic Models, Department of Molecular Neuropharmacology, Institute of Pharmacology, Polish Academy of Sciences, Smętna 12, 31-343, Krakow, Poland; 2 Department of Neurophysiology and Chronobiology, Institute of Zoology, Jagiellonian University, Gronostajowa 9, 30-387 Krakow, Poland; 3 Cell Biology, Department of Clinical and Experimental Medicine, Linköping University, SE-581 85, Linköping, Sweden

## Abstract

The role of changes in dopamine neuronal activity during the development of symptoms in affective disorders remains controversial. Here, we show that inactivation of NMDA receptors on dopaminergic neurons in adult mice led to the development of affective disorder-like symptoms. The loss of NMDA receptors altered activity and caused complete NMDA-insensitivity in dopamine-like neurons. Mutant mice exhibited increased immobility in the forced swim test and a decrease in social interactions. Mutation also led to reduced saccharin intake, however the preference of sweet taste was not significantly decreased. Additionally, we found that while mutant mice were slower to learn instrumental tasks, they were able to reach the same performance levels, had normal sensitivity to feedback and showed similar motivation to exert effort as control animals. Taken together these results show that inducing the loss of NMDA receptor-dependent activity in dopamine neurons is associated with development of affective disorder-like symptoms.

Affective disorders are associated with dysfunctions in reward processing, which manifest as anhedonia, altered sensitivity to reinforcement, a reduced ability to exert effort or decreased incentive motivation^1^,^2^,^3^. These functions depend on the brain’s reward system and are mainly mediated by dopamine signalling via the mesolimbic and corticolimbic pathways^4^,^5^. Nevertheless, the role that dopamine signalling plays in the development of affective disorders is often regarded as secondary to other monoamines^6^,^7^,^8^,^9^.

Evidence supporting the notion that dopamine signalling strongly contributes to the development of affective disorders comes from both animal model studies and clinical studies. Clinical studies have shown that pharmacotherapies that mainly target dopamine reuptake may be as effective as selective serotonin and/or noradrenaline reuptake inhibitors^10^,^11^,^12^. Accordingly, it was observed in animal models that treatment with antidepressant drugs either directly or indirectly affected dopamine signalling in the basal ganglia^13^,^14^. Furthermore, in Flinders sensitive rats, which are a classic animal model of depression^15^, depressive-like symptoms were correlated with reduced burst firing in dopamine neurons and lower dopamine levels in the nucleus accumbens septi (NAc)^13^,^16^. In line with these data, it has also been observed that chronic mild stress procedures, which lead to the development of symptoms that are associated with depression, reduced the activity of dopamine neurons^17^. Moreover, in mice with halorhodopsin-mediated inhibition of dopamine neuronal activity resulted in behavioural despair-like immobility in forced swim test, decreased sucrose preference^18^ and reduced social interactions^19^. Another study that used a similar model to induce channelrhodopsin-activated burst-like activity in VTA dopamine neurons showed that the increase in activity caused greater susceptibility to the effects of social defeat, which may indicate that dopamine neurons play a specific role in the underlying cause of depressive-like symptoms^20^.

Taken together, these studies suggest the possibility that dopamine signalling, especially altered burst activity in midbrain dopaminergic neurons (DA neurons), may underlie the development of at least some affective disorder symptoms. Phasic activity is mainly controlled by NMDA receptors^21^,^22^ and it was previously reported that in mice with inactivation of NMDA receptors on DA neurons bursting is significantly attenuated^23^,^24^. Here we use a mouse model with inducible inactivation of NMDA receptors in dopamine neurons to test whether the mutation leads to development of symptoms related to affective disorders.

## Methods

### Animals

All behavioural procedures were approved by the II Local Bioethics Committee in Krakow (permit number 1000/2012, issued on November 26, 2012 and 957/2012 issued on July 26, 2012 and number ZI/819/2013 issued on November 1, 2013) and conducted in accordance with the European Communities Council Directive of 24 November 1986 (86/609/EEC). Animals were housed 2–5 per cage in rooms with a controlled temperature of 22 ± 2 °C under a 12/12 h light-dark cycle with *ad libitum* access to food and water. The mice were treated with tamoxifen (Sigma, Poznan, Poland) when they reached 8–10 weeks old. Prior to injection, the tamoxifen was dissolved in sunflower oil and filtered through a 0.22-μm membrane. Injections were performed once daily for 5 days. The tamoxifen dose was 100 mg/kg and was injected at 5 μl/g. The mice were allowed to rest for at least 3 weeks before the experiments were initiated. The control mice were tamoxifen-injected (0/0; flox/flox) mice and mutants were tamoxifen-injected NR1^DATCreERT2^ (Tg/0; flox/flox) animals. Physiological recordings and behavioural tests were performed on male mice, with the exception of the IntelliCage experiments, which were performed on females.

### Electrophysiology

The electrophysiological experiments were performed using 9 mutant and 8 control mice that were anesthetized with urethane. Neuronal activity was extracellularly recorded, and DA cells were identified based on electrophysiological criteria^25^,^26^. The responses of DA-like neurons to NMDA were tested following the iontophoretic application of the drug. All technical details are described in the Supplemental methods.

### Forced swim test

The animals were placed in a beaker that was 13 cm in diameter and 35 cm high that was filled up to ~14.5 cm with 2 l of water at 28 °C). Mouse behaviours were recorded for 6 minutes, and activity during the test was analysed using EthoVision XT software, version 8 (Noldus, Netherlands).

### Social interaction

The tests were performed in a plastic cage (20.5 × 55 × 38.5 cm) that was illuminated at 50 lux. Behaviours were recorded using a camera that was placed above the cage (DMK 22AUC03, The Imaging Source, Bremen, Germany). First, the experimental mouse was placed in the cage for 30 minutes, after which a new mouse was introduced to the cage for 10 minutes. After the interaction sessions were completed, the video recordings were analysed using EthoVision XT software version 11.5. For each trial, we verified that animal positions were automatically detected and manually corrected the results when necessary. For the purpose of analysing distance between mice, “close proximity” was defined as less than 5 cm between body centres.

### Saccharin preference

Male mice were tested individually in cages for 24 h where they had access to two 25 ml graduated drinking tubes. One tube was filled with water and the other with 0.1% saccharin solution. Food was provided ad libitum on the cage floor.

Saccharin preference with effort discounting was tested in an IntelliCage (New Behavior, Zurich, Switzerland), which allows the tracking of behaviours in animals that have been implanted with RFID chips (UNO PICO ID, AnimaLab, Poznań, Poland). Female mice were used in the experiment to avoid aggressive behaviours. First simple saccharin preference was tested. Mice were placed in the cage for 4 days, with free access to water. On the 5^th^ day bottles containing a 0.1% saccharin solution were placed in two of the corners. The guillotine doors were closed, but when the animal explored the door (detected by a photo cell) the doors would open for 10 s. A separate cohort of mice was tested under conditions where access to saccharin was increasingly more difficult. The animals were first allowed to adapt to the cage for 72 h, followed by free access to saccharin or water for another 72 h. Then, access to saccharin required approaching the guillotine door a progressively higher number of times (1, 3, 5, 7, 9 or 11 times), with an increase after 48 hours. The positions of the bottles were exchanged after each 24 h interval.

### Instrumental food self-administration

The food self-administration procedure was performed in operant conditioning chambers (ENV-307W, Med Associates). Before the start of the procedure, the mice were food-restricted to maintain 80–85% of their initial weights. In our experience this level of restriction is sufficient to avoid reduction of responding by the end of self-administration session due to satiety. Mean weight before restriction was 27.18 ± 0.69 g and it dropped to 23.52 ± 0.67 g before the first session. Food self-administration sessions lasted 45 minutes. An instrumental response (a nose poke) on the active operand resulted in the delivery of a food pellet (20 mg, BioServ, Flemington, NJ, United States) whereas a response on the inactive operand had no consequences. After 10 days of food self-administration, the animals were introduced to a progressive ratio (PR). Under PR schedule after each food pellet was delivered, the number of instrumental responses that was required to receive a pellet was increased. The mice were first tested under a PR1, and they were tested under a PR3 on the subsequent day.

### Effort discounting in a T-maze task

The procedure was based on refs 27,28 and was adjusted for mice. The animals were food-restricted and maintained at 80–85% of their initial weight. Mean weight before restriction was 26.15 ± 0.45 g and it dropped to 22.84 ± 0.48 g before the animals were introduced to the T-maze apparatus. After mice were allowed to habituate to the apparatus (3 days), testing started, and 12 trials were run every day. Each animal was assigned a large and small reward arm that contained 4 or 1 chocolate food pellets, respectively (20 mg, Chocolate Flavor, BioServ). The larger reward was placed in the arm that was less preferred during habituation. During the first 5 days of testing, a choice was forced by allowing access to only one of the reward arms. Then, the animals were allowed to freely choose which arm to explore. After 5 sessions, an obstacle that was 5 cm high was placed in the arm with 4 pellets, and then after each subsequent 5 sessions the size of the obstacle was increased sequentially to 8, 12 and 14 cm. Access to the smaller reward remained free. Trials during which the mouse entered the arm with large reward but did not cross the barrier were repeated.

### Probabilistic reversal learning task

The procedure was based on the protocol described in ref. 29. Briefly, food-restricted animals (mean weight before restriction 27.37 ± 0.54 g and 23.13 ± 0.59 g before first session) underwent training during which they learned to nose poke a highlighted active operand to obtain a food reward. Trials were initiated to allow exploration of the food receptacle, and during these trials, the left or right cue-light was lit in a pseudorandom manner. Once the pre-determined criterion of 30 rewarded responses during two consecutive days was reached, the animals were introduced to a probabilistic reversal learning (PRL) task. During PRL, the cue-lights in both nose poke operands were lit, and the animals were free to choose between the operands, with the probability of a reward delivery of 0.8 or 0.2. Over 15 sessions, the probabilities that were assigned to each operand were constant between the sessions until an animal acquired a criterion of choosing the side with the higher reward probability 90% of the time. The sides were reversed with each consecutive session once this criterion had been met. The sessions lasted 40 minutes.

### Data analysis

Statistical analyses were conducted using GraphPad Prism and R software. Statistical significance was estimated using analysis of variance (ANOVA) followed by a post-hoc test where appropriate. Tukey’s range test was used as the default post-hoc test, Bonferroni corrected t-test was used as post-hoc in cases where mixed-design ANOVA was applied. Comparisons between two samples were performed using Student’s t tests or nonparametric tests.

## Results

### The effect of NR1 inactivation on spontaneous and NMDA-induced activity in DA-like neurons

The NR1^DATCreERT2^ strain was generated by crossing DATCreERT2 mice^30^ with a strain carrying a floxed variant of the NR1 (*Grin1*) gene^31^. The expression of the CreERT2 is driven by the promoter of the DAT gene, which selectively targets the mutation to dopamine neurons. The recombinase remains in the cytosol until the mouse is treated with tamoxifen, which enables translocation of the recombinase to the nucleus and recombination of the target sequence^32^. The DATCreERT2 transgene was previously confirmed to have high efficiency and specificity in inducing gene recombination^30^,^33^,^34^.

**Figure 1:**
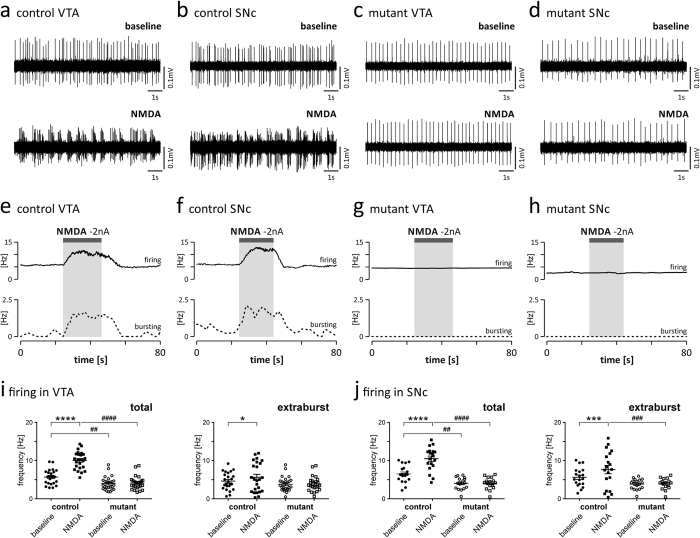
The control and NR1^DATCreERT2^ mice differ in respect to spontaneous and NMDA-induced firing of DA-like neurons in VTA and SNc. Panels (a,b,c and d) show fragments of raw recordings from four different DA-like cells located in VTA and SNc of control (**a,b**) and mutant (**c,d**) animal. The top traces were recorded under baseline conditions and the bottom traces show activity after the iontophoretic application of NMDA. Note the NMDA induced change in the pattern of firing observed in VTA and SNc of control animal (**a,b**) and lack of response in NR1^DATCreERT2^ animal (**c,d**). Firing and bursting rate histograms, generated for the same example DA-like neurons are shown on panels (e,f,g and h). The grey horizontal bars and shaded areas indicate the time at which NMDA was applied. Once again note the clear, NMDA induced, increase in the rate of firing (top) and bursting (bottom) of DA-like neuron in VTA and SNc of control animal (**e,f**) and lack of any change in NR1^DATCreERT2^ animal (**g,h**). Graphs in panels (i,j) show the total and extra burst firing rates of all recorded DA-like neurons in the VTA (**i**) and SNc (**j**) of control and mutant mice observed under baseline conditions and after the iontophoretic application of NMDA. Control animals: ● - baseline, ■ - NMDA; Mutant animals: ○ - baseline, □ - NMDA. Horizontal lines with whiskers indicate the mean and SEM of a parameter. Parameter values and statistics are shown in Supplementary Tables S2 and S3. ‘*’, indicates Bonferroni corrected t-test that resulted in p < 0.05, ‘**’ indicates p < 0.01, ‘***’ indicates p < 0.001 and ‘****’ indicates p < 0.0001.

Electrophysiological experiments were performed on naïve mice 4 weeks after they were treated with tamoxifen. A total of 133 neurons were recorded from the midbrains of 9 control and 8 mutant animals. We used electrophysiological criteria^25^,^26^, pharmacological testing and anatomical localization to classify 100 of these cells as DA-like neurons (Supplementary Figures S1 and S2). These 100 neurons were further split into two groups: VTA (34 control and 26 mutant neurons) and SNc (22 control and 18 mutant neurons). We found that in the mutant animals, both the VTA and the SNc neurons had significantly lower total firing rates than the controls under baseline conditions (Supplementary Table S1). Additionally, extra burst firing rates were significantly lower in the DA-like neurons that were localized in the SNc in the mutant mice than in the corresponding neurons in the control mice (Supplementary Table S1). Next, we tested the responses of DA-like neurons to iontophoretically applied NMDA. In the control animals, NMDA induced an increase in total firing rate in all of the DA-like neurons. Moreover, applying NMDA significantly increased the extra burst firing rate in the VTA and SNc in the control mice. In contrast, NMDA had no effect on the total or extra burst firing rate in the mutant animals (Fig. 1; Supplementary Tables S2 and S3).

**Figure 2:**
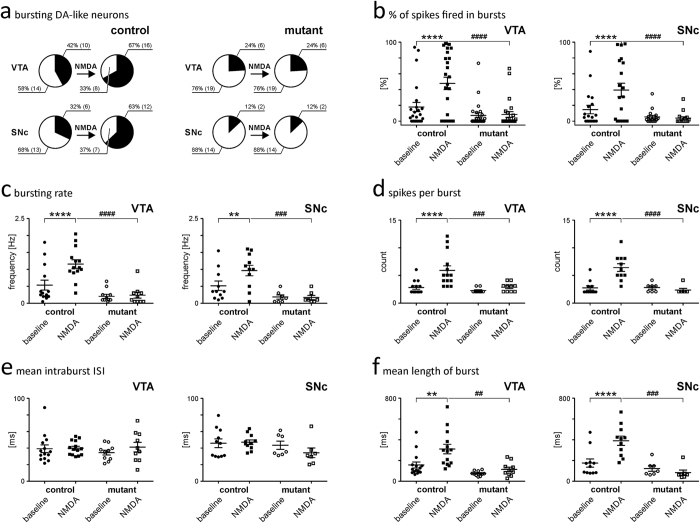
Iontophoretic application of NMDA enhances bursting in DA-like neurons in the VTA and SNc in control but not NR1^DATCreERT2^ mice. (**a**) Pie charts showing that NMDA induced an increase in the percentage of bursting DA-like neurons in the VTA and SNc of control animals, but a complete lack of NMDA-induced change in the proportion of bursting DA-like neurons in mutant mice. The numbers of neurons that were recorded are shown in brackets. (**b–f**) Graphs showing that NMDA induced an increase in most of the burst parameters that were generated by DA-like neurons in control animals, while NMDA induced no changes in burst parameters in mutant mice. Control animals: ● - baseline, ■ - NMDA; Mutant animals: ○ - baseline, □ - NMDA. Horizontal lines with whiskers indicate the mean and SEM of a parameter. Parameter values and statistics are shown in Supplementary Tables S4 and S5. ‘**’ Indicates Bonferroni’s multiple comparisons tests that resulted in p < 0.01, ‘***’ indicates p < 0.001 and ‘****’ indicates p < 0.0001.

In all control mice, we observed an increase in firing rates after NMDA was applied. This increase was accompanied by an increase in the proportion of DA-like neurons that expressed a bursting pattern of electrical activity (Fig. 2a) and a significant increase in the percentage of spikes that were fired in bursts (Fig. 2b; Supplementary Tables S4 and S5). An analysis of burst parameters revealed that the NMDA induced an increase in the bursting rate and the number of spikes per burst in addition to extending the length of each individual burst without affecting interspike intervals (Fig. 2c–f, Supplementary Tables S4 and S5). In contrast in the mutant mice, NMDA did not change or affect the pattern of activity of DA-like neurons (Fig. 2a and b; Supplementary Tables S4 and S5). There were no significant differences in the parameters of baseline bursting of control and mutant animals (Fig. 2b–f). As expected, in the NR1^DATCreERT2^ mice, we didn’t encounter any NMDA-sensitive DA-like, while all non-DA-like neurons showed changes in activity following the application of NMDA (Supplementary Figure S3).

Importantly, we did not observe a reduction in firing of DA-like neurons in mutant mice that could had resulted from activation of neighbouring GABAergic cells. While application of a 2 nA current led to release of NMDA that was sufficient to elicit robust responses from control DA-like neurons, a 5–10 nA current was required in case of non-DA-like cells (Figures S2 and S3). The iontophoretic release is highly localized and the effective NMDA dose reaching non-targeted cells was much lower.

No recruitment of silent non-DA cells while applying NMDA to DA-like neurons was observed.

### Behavioural despair and social interaction

**Figure 3:**
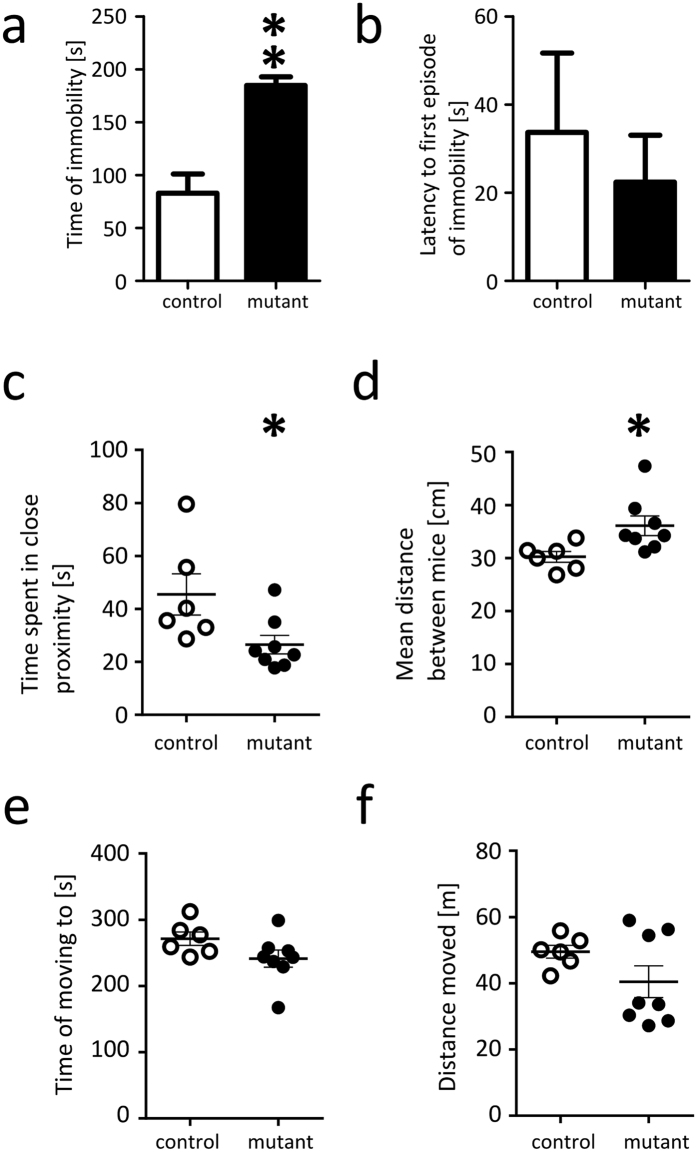
NR1^DATCreERT2^ mice exhibit impairments in forced swim and social interaction tests. (**a,b**) Behaviour in forced swim test. (**a**) Bars show the mean time the animals were immobile during the 6-minute test (Mann-Whitney test, p < 0.01). (**b**) Time to the first immobility episode in the forced swim test (Mann-Whitney, ns). (**c–f**) Social interaction test. Mutant mice were placed in the test box for 30 minutes, after which a second mouse was introduced for 10 minutes. (**c**) Relative amount of time spent in close proximity (body centres < 5 cm apart; Mann-Whitney test, p < 0.05). (**d**) Mean distance between the animals during the test (Mann-Whitney test, p < 0.05). (**e**) Relative amount of time the mouse spent moving towards the interaction partner (Mann-Whitney test, ns). (**f**) Relative total distance moved during the social interaction test (Mann-Whitney test, ns). Group sizes were 7 control & 7 mutant male mice in the forced swim test, and 6 controls & 8 mutants in the social interaction test. Error bars show the s.e.m., ‘*’, indicates Mann-Whitney tests that resulted in p < 0.05, ‘**’ indicates p < 0.01, and ‘***’ indicates p < 0.001.

First, we tested the NR1^DATCreERT2^ mice in forced swim test, in which increased immobility is interpreted as an indicator of behavioural despair. The mutant mice spent significantly more time immobile than control animals (Fig. 3a). No difference was observed in the latency to the first episode of immobility (Fig. 3b). Next, we tested non-aggressive social interactions with a non-familiar conspecific in a separate cohort of mice. The NR1^DATCreERT2^ mice spent less time in close proximity to and maintained a greater distance from the interaction partner (Fig. 3c) during the 10 minute interaction test (Fig. 3d). A trend towards a shorter time to move in the direction of the interaction partner was observed but was not statistically significant (Fig. 3e). The difference in distance moved during the 10 minute interaction period was not significant (Fig. 3f). An additional replication cohort was tested to validate these results. The experiment confirmed that the mutation caused a decrease in time spent in close proximity and additionally showed a significant effect on the distance travelled during the test (Figure S4). To exclude a mutation-induced motor impairment effect, we performed a rotarod test that showed that the mutation had no effect on the latency to fall (Supplementary Figure S5). Together, these experiments indicate that in dopamine neurons, the loss of NMDA receptor-dependent activity results in increased immobility during forced swim test and a reduced amount of time spent in social interactions.

### Saccharin preference

**Figure 4:**
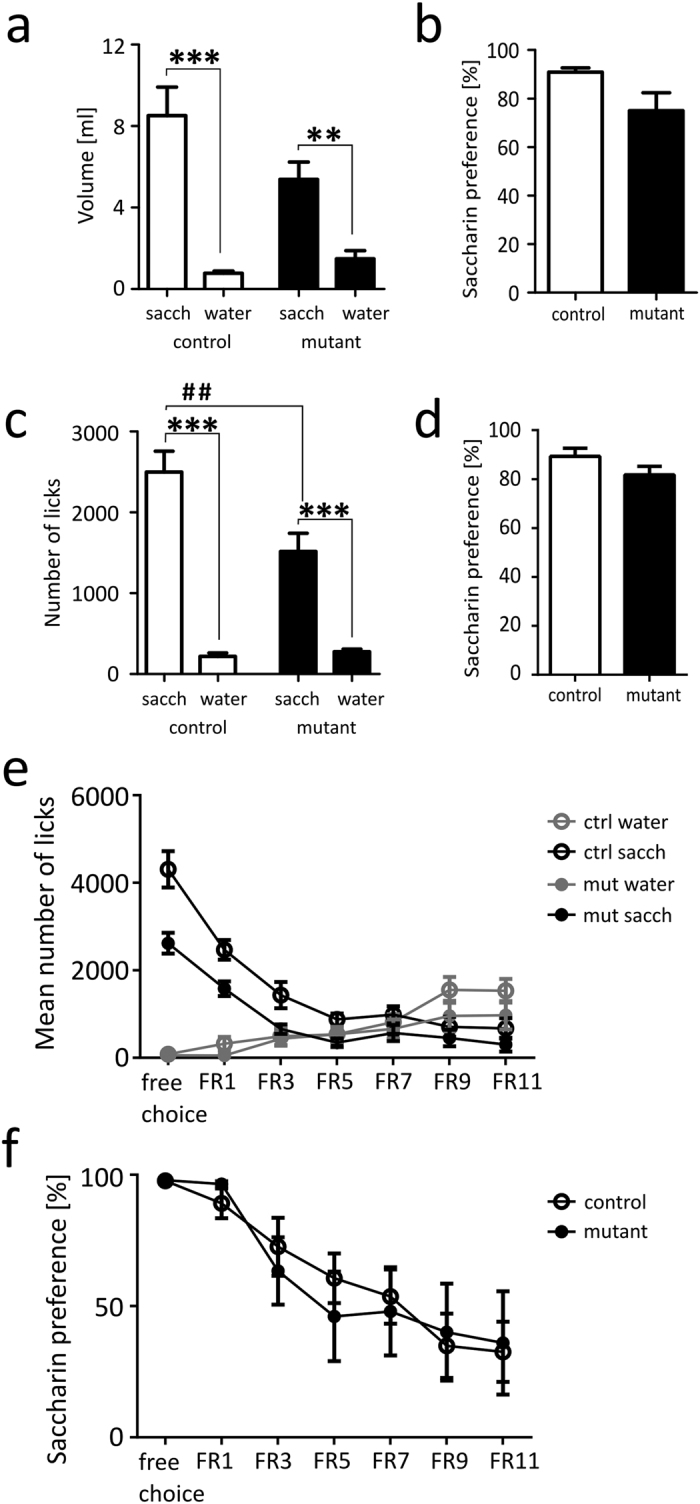
Saccharin preference tests and effort-based learning in an IntelliCage. (**a**) The amount of saccharin and water drunk during 24 h saccharin preference test. (**b**) Saccharin preference in 24 h experiment in individual cages (Mann-Whitney, ns). (**c–f**) Experiments in an IntelliCage (**c**) Mean number of licks performed by animals in the saccharin and water corners under a fixed ratio 1 schedule. (**d**) Saccharin preference under a fixed ratio 1 schedule (ns). (**e**) Mean number of licks during effort-based test in an IntelliCage. (**f**) Saccharin preference during consecutive schedules of effort-based task. Group sizes were 7 controls & 8 mutant male mice in saccharin preference, 10 control & 13 mutant female mice in the saccharin preference test in the IntelliCage and 6 control & 8 mutant female mice in the effort-based test. Error bars show the s.e.m., ‘*’ indicates post-hoc test p < 0.05, ‘**’ or ^‘##’^ indicates p < 0.01, and ‘***’ indicates p < 0.001.

First we tested sweet-taste preference in male mice that were placed in individual cages and offered choice between 0.1% saccharin or water for 24 h. Both control and mutant mice drank more saccharin than water, however analysis indicated an interaction between genotype and intake (Fig. 4a, two-way ANOVA, *genotype*, F_1,26_ = 2.139, p = 0.1556; *drink*, F_1,26_ = 47.928, p < 0.0001, *genotype x drink*, F_1,26_ = 5.435, p = 0.0278), a trend towards lower saccharin intake in mutant mice (Fig. 4a, p = 0.0569) and also a trend towards lower saccharin preference (Fig. 4b, p = 0.0719). In order to validate a possible effect of the mutation on saccharin intake we have performed a series of experiments with extended access to saccharin and without social isolation using female mice in IntelliCages. Control and mutant female mice were first habituated to the cage for 4 days, during which their activity gradually decreased from an initial peak (Supplementary Figure S6a, two-way ANOVA, *genotype*, F_1,22_ = 3.936, p = 0.0599; *time*, F_3,66_ = 55.07, p < 0.0001; *genotype x time*, F_3,66_ = 1.631, p = 0.1905). When mice were given access to 0.1% saccharin, both the control and the mutant mice more frequently visited the corners with access to saccharin (Supplementary Figure S6b). The mutation had a significant effect on the total number of visits (two-way ANOVA, *genotype*, F_1,42_ = 54.59, p < 0.0001; *drink*, F_1,42_ = 41.52, p < 0.0001; *genotype x drink*, F_1,42_ = 4.39, p = 0.0423). Moreover, while the mutant mice performed fewer licks on the saccharin bottles (Fig. 4c, two-way ANOVA, *genotype*, F_1,44_ = 6.82, p = 0.0123; *drink*, F_1,44_ = 98.62, p < 0.0001; *genotype x drink*, F_1,44_ = 8.610, p = 0.0053), there was no difference in the mean number of licks on the water bottles. Both the *NR1*^*DATCreERT*2^ and the control mice preferred to drink saccharin over water (Fig. 4d). Additionally, the mutation had a significant effect on the fraction of corner visits during which the animals were observed to drink (Supplementary Figure S6c; two-way ANOVA, *genotype*, F_1,42_ = 6.789, p = 0.0126; *drink*, F_1,42_ = 79.35, p < 0.0001; *genotype x drink*, F_1,42_ = 0.9291, p = 0.3406).

To determine whether the lower intake of saccharin was caused by greater effort discounting, we tested another cohort of female mice using a schedule in which it became progressively more difficult to access saccharin. We found a significant effect of the mutation on the number of visits to the corners (Supplementary Figure S6d; significant effects in three-way repeated measures ANOVA, *genotype*, F_1,14_ = 7.304, p = 0.0172, *difficulty*, F_6,84_ = 32.661, p < 0.0001; *drink*, F_1,98_ = 87.102, p < 0.0001, *difficulty x drink*, F_6,98_ = 6.939, p < 0.0001) and the number of licks that were performed on the saccharin bottles (Fig. 4e; significant effects in three-way repeated measures ANOVA, *genotype*, F_1,14_ = 32.1, p < 0.0001; *difficulty*, F_6,84_ = 39.188, p < 0.0001; *drink*, F_1,98_ = 40.694, p < 0.0001; *difficulty x drink*, F_6,98_ = 29.378, p < 0.0001; *genotype x drink*, F_1,98_ = 4.244, p = 0.042) but not on saccharin preference. We did not observe an interaction between access difficulty and preference (Fig. 4f).

### Motivation and effort

**Figure 5:**
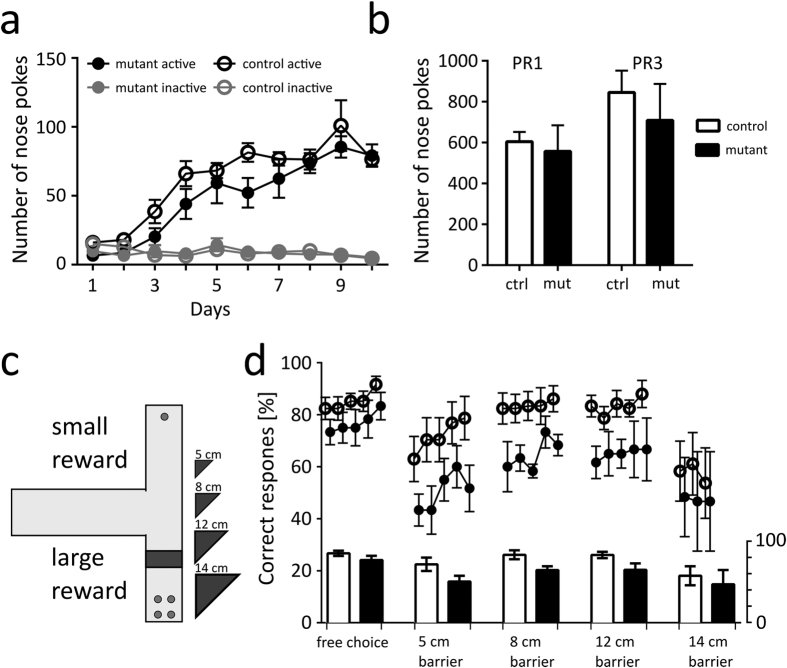
Instrumental responding in Skinner boxes and effort-based T-maze test. (**a**) Food self-administration under a fixed ratio 1 (FR1) schedule. The graph shows the mean numbers of nose pokes over 10 test sessions. (**b**) Instrumental responding under a progressive ratio schedule. Bars show the mean number of nose pokes the animals performed under a progressive ratio schedule of 1 and 3 in which the mice had to increase their instrumental response by 1 or 3, respectively, to obtain food pellets. (**c**) A diagram summarizing the effort-based T-maze experiment. The animals were first trained to learn about two possible rewards, and they were then offered a choice between them. During the experiment, access to the larger reward was initially free but was then barred by increasingly larger obstacles. (**d**) The graphs show preference of the larger reward on each session, and the bars below show the mean preference during each stage of the experiment. Group sizes were 7 control & 9 mutant male mice in the experiments involving instrumental food self-administration (**a,b**) and 7 control & 5 mutant male mice in the T-maze task. Error bars show the s.e.m.

We found that the mutant mice were slower to learn instrumental food self-administration, but they reached the same response plateau after 10 sessions (Fig. 5a; significant effects in three-way repeated measures ANOVA, *genotype x active/inactive*, F_1,140_ = 9.858, p = 0.002; *time* × *active/inactive*, F_9,140_ = 23.197, p < 0.0001; *time*, F_9,126_ = 23.273, p < 0.0001; *active/inactive*, F_1,140_ = 600.025, p < 0.0001). Then, the animals were tested under a progressive ratio reinforcement schedule (PR1 and PR3) wherein the number of responses that were performed was regarded as a measure of motivation to obtain a reward (Fig. 5b)^35^,^36^,^37^. Under either test condition, no genotype-dependent differences were observed in the number of instrumental responses that were performed.

To further determine whether the mutation affected effort discounting, we used a T-maze task wherein the mice could choose between a small and freely accessible reward or a large reward behind an obstacle (Fig. 5c). Initially, the mice received equal access to each maze arm, and they showed a preference for the larger reward (85.4% and 77% in the control and mutant mice, respectively). Then, a barrier was placed on the path to the larger reward that the animal was required to climb across to reach the reward. We found that the presence of barriers of increasing height resulted in a decrease in the preference for the larger reward in both the control and the mutant mice (Fig. 5d; two-way ANOVA, *time*, F_22,264_ = 6.150, p < 0.0001). Although the mutant animals did not perform significantly different from the controls in the T-maze test, there was a general trend toward a lower preference for the larger reward (Fig. 5d; two-way ANOVA, *genotype*, F_1,12_ = 3.444, p = 0.0882, *genotype* × *time*, F_22,264_ = 0.5933, p = 0.9269).

### Cognitive flexibility and sensitivity to negative feedback

We used a probabilistic reversal learning (PRL) task to assess changes in cognitive flexibility and the processing of positive and negative feedback. In these tasks, the animals first learned to choose between two instrumental responses with different probabilities of yielding a reward (20 or 80%). They were then tested to determine their ability to adapt to reversals of reward probabilities. We observed that the mutant mice required a significantly higher number of sessions to reach the performance criterion during the training and initial discrimination phase, but they were not different from the control animals after the first reward reversal (Supplementary Figure 7a and 7b). There was no significant difference between the mutant and the control mice in their sensitivity to positive and negative feedback based on the frequencies with which they selected the same side when it was previously rewarded or shifted to the other side after no reward was received (Supplementary Figure 7c and 7d). The mutation did not affect the number of completed trials or reversals, and it had no effect on trial duration, the latency to respond or the latency to collect a reward from the food receptacle (Supplementary Table S6). Taken together, the mutation had no significant effect on positive or negative feedback sensitivity.

## Discussion

We found that when the loss of the NR1 subunit was specifically induced in DA neurons in adult mice, there were alterations in the pattern of electrical activity that led to changes in selected behaviours known to be associated with affective disorder symptoms. In addition to confirming the loss of functional NMDA receptors in DA-like neurons in the mutant mice, our analyses of neuronal activity led to some interesting observations. The mutation did not completely abolish the ability of some DA neurons to generate bursts of action potentials. In most cases, the bursts that were recorded in the *NR1*^*DATCreERT2*^ mice were doublets (i.e., two spikes within 80 ms), which are the most common type of spontaneous bursts^22^,^38^. The fact that the ability to generate doublets was preserved in small fraction of DA neurons in mutant mice indicates that firing two-spike bursts relies on mechanisms that are independent of NMDA receptors. Interestingly, it has been shown that a similar proportion of DA-like neurons can generate bursts of action potentials in response to cholinergic receptor activation^39^. It was previously reported that the constitutive loss of NMDA receptors during early development reduces the total firing rate of DA-like neurons without influencing the extraburst firing frequency^23^,^24^. Our results confirm these findings in VTA neurons but not in SNc DA-like neurons, in which basal extra burst activity was significantly reduced in the mutant mice. A similar effect was not reported by previous studies that were based primarily on VTA recordings^23^,^24^. In general, the direction of changes that was caused by the mutation and the magnitude of these changes were in agreement with previous studies that used mutant mice or the iontophoretic application of NMDA receptor antagonists^21^,^23^,^24^,^38^,^40^. Nevertheless, the differences that have been observed between the activity of DA-like neurons in mutants with inducible and constitutive mutations are potentially relevant to behaviour because their phenotypes differ considerably.

Inactivating the NMDA receptors on dopamine neurons led to the development of behaviours that are associated with affective disorders. The mutant mice showed more immobility in forced swim test and a decreased propensity for social interaction. These behaviours are considered to be indicators of despair and impaired social function and are associated with affective disorders^41^. Our results are consistent with those of previous studies that have reported a similar phenotype in mice following the optogenetic inhibition of DA neurons^18^,^19^, but our results differ from the findings in a study of mice with a constitutive mutation in the NR1 subunit in dopamine neurons^23^. We attribute this difference to the development of compensatory changes in mice with non-inducible mutations, such as the reported increase in frequency of spontaneous EPSCs in dopamine neurons^30^. The possibility that the observed changes in behaviour were caused by impaired motor functions is unlikely because the mutant mice did not show any impairments in rotarod performance. Moreover, it was previously shown that *NR1*^*DATCreERT2*^ mice had normal activity in an open field^30^ and no differences in open field behaviour were reported in four different *NR1*^*DATCre*^ strains^24^,^30^,^42^,^43^. In case of one strain a decrease in horizontal activity during initial exploration of a novel environment was observed^44^. We speculate that decreased activity during the social interaction test may be related to a possible increase in anxiety-like behaviours, as was reported in case of one of the *NR1*^*DATCre*^ strains^45^.

The results of saccharin preference experiments showed that there was no evidence that the mutation caused anhedonia, defined as a reduced ability to experience pleasure. It is interesting to consider the observed reduction in saccharin intake in the context of the consummatory and motivational aspects of anhedonia in depressive patients, as defined by Treadway and Zald^2^. We observed no evidence of consummatory anhedonia; however, altered component of motivational anhedonia may be a plausible explanation for the reduction we observed in the amount of saccharin that was consumed. Mutant mice had similar ability to exert effort as controls, but were less likely to engage in the behaviour. In the IntelliCage experiment *NR1*^*DATCerER2*^mice made fewer visits to the corners where water or the saccharin solution were available, however this had no significant effect on preference of saccharin over water. Our observation is consistent with the notion the activity of DA neurons encodes incentive salience, or “wanting” the reward rather than “liking” the reward^46^. We note however that our results differ from the effects on sucrose preference that were reported in another study following the optogenetic inhibition of dopamine neurons. In that study, a significant decrease was reported in sucrose preference^18^.

The fact that the mutation had no significant effect on effort discounting was an unexpected observation, considering the established role of the mesolimbic system in motivation^47^,^48^. While the NR1^DATCreERT2^ mice were slower to learn instrumental responses, which is consistent with other studies^42^,^43^, they were overall not significantly different in the effort they exerted under a progressive ratio instrumental schedule or in the T-maze task. In case of the progressive ratio schedule task it should be noted that nose poking has lower kinetic requirement than lever pressing and thus could be less affected by effort discounting^49^. Conversely, there was a trend towards greater discounting of effort by the mutant mice in the T-maze procedure. This again suggests that the decrease in saccharin consumption could have been caused by a decrease in the probability of initiating the behaviour that led to the consumption of a reward rather than insufficient vigour for completing it. Finally, we observed no significant difference in lose-shift behaviour or the number of completed reversals in the probabilistic reversal learning task, and there was therefore no indication of an increase in sensitivity to negative feedback or impaired cognitive flexibility.

Taken together, our results and those described in other reports agree that decreases in the activity of the DA neurons in the ventral midbrain leads to changes in behaviours that resemble the symptoms of affective disorders. Moreover, the reverse may also be true, in that procedures that cause a depressive-like phenotype in rodents also cause a decrease in the activity of midbrain DA neurons. Importantly, our data show that at a molecular level, loss of NMDA receptor-dependent activity, and most probably its effect on burst activity, was responsible for specific changes in behaviour, including despair-like behaviours, reduced social activity and reduced saccharin intake. However, these changes were not associated with significantly altered sensitivity to reinforcement, and the results indicated only a trend toward a reduced motivation to initiate action or greater effort discounting.

## Additional Information

**How to cite this article**: Jastrzębska, K. *et al.* Loss of NMDA receptors in dopamine neurons leads to the development of affective disorder-like symptoms in mice. *Sci. Rep.* **6**, 37171; doi: 10.1038/srep37171 (2016).

**Publisher’s note:** Springer Nature remains neutral with regard to jurisdictional claims in published maps and institutional affiliations.

## Supplementary Material

Supplementary Information
