# Supplementary material for: Loss of NMDA receptors in dopamine neurons leads to the development of affective disorder-like symptoms in mice
Source: Sci Rep. 2016 Nov 17;6:37171. doi: 10.1038/srep37171 (PMC5112557; doi:10.1038/srep37171)
Supplement: Supplementary Information [file srep37171-s1.pdf]

Loss of NMDA receptors in dopamine neurons leads to the development of affective disorder-like symptoms in mice

Kamila Jastrzębska[1#], Magdalena Walczak[2#], Przemysław Eligiusz Cieślak[1], Łukasz Szumiec[1], Mateusz Turbasa[1], David Engblom[3], Tomasz Błasiak[2], Jan Rodriguez Parkitna[1*]

Supplementary Methods

Electrophysiology

Surgical procedure. Mouse body temperatures were monitored using an automatic heating pad (temperature controller TCP-02, WMT) and maintained at 37°C. Animals were mounted in a stereotaxic frame (SF-1450AP, ASI Instruments Inc.; Warren, MI, USA) on standard ear (EB-918, 18° tip) and incisor bars (RA-200). Recording electrodes were positioned in the VTA/SNc using the following stereotaxic coordinates: 3.1 - 3.2 mm caudal from bregma, 0.7 - 0.9 mm lateral from the middle of the sagittal sinus, 4.5 - 5 mm ventral from the skull surface at bregma point[1].

Extracellular recording and iontophoretic drug application. Extracellular recordings were performed using 3-barrel microiontophoretic pipettes that were prepared from borosilicate glass capillaries (o.d.: 1 mm, i.d.: 0.78 mm, with internal filament; Sutter Instruments, USA) and pulled using a vertical puller (model PE-21, Narishinge International Instruments, Japan). The recording pipette was filled with 2% Chicago Sky Blue in 2 M NaCl (impedance 7-10 MΩ at 1000 kHz). The surrounding barrels were filled with NMDA (100 mM in 0.9% NaCl, pH 8) and 0.9% NaCl (current balancing pipette). A negative current in the range of 2-5 nA was used for the NMDA ejections and a positive current in the range of 10-15 nA was used to retain the substances in the pipettes. All applications were performed using a 2-channel iontophoresis amplifier with an automatic balance (MVCS-02C, NPI electronic GmbH, Germany). The timing of the application was controlled using a 1401 mkII interface. Signals were amplified (10 000x)

and filtered (300-5000 Hz) using an A-M Systems microelectrode amplifier (model 1800; Carlsborg, WA, USA). Analogue signals were digitized for storage and further analysis using a Micro 1401 mkII interface equipped with Spike2 software (Cambridge Electronic Design Inc.; Cambridge, UK). After the end of the recordings, a negative current of 15 μA (isolated pulse stimulator; model 2100; A-M Systems; Carlsborg, WA, USA) was passed through the recording pipette for 15 minutes to deposit a dye to mark at the recording site.

Identification of dopamine neurons. Neurons were classified as dopamine-like when they met the following previously established criteria[2]: broad (>1.1 ms), triphasic action potential (length measured from AP initiation to the minimum of the trough) and a firing rate below 10 Hz (Supplementary Figure 1c). Additionally, a portion of the recorded neurons were tested using a D2 receptor agonist (quinpirole, 0.5 mg/kg, diluted in saline, Tocris, Bristol, United Kingdom) and antagonist (eticlopride, 5 mg/kg, diluted in saline, Tocris, Bristol, United Kingdom, Supplementary Figure S2). The final classification of the neurons that were DA-like was performed after a histological confirmation of the recording sites within the VTA or SNc (Supplementary Figure 1a, b).

Histology. At the end of the experiments, the animals were sacrificed by decapitation, and their brains were extracted, fixed in 4% formaldehyde in 0.1 M phosphate buffer (pH 7.2) for at least 48 hours and then sliced at 50 μm in the coronal plane using a microtome (Leica VT1000S, Heidelberg, Germany). Slices containing dye deposits were photographed to verify the placement of the recording with reference to a stereotaxic atlas of the mouse brain[1]. The positions of all of the recorded DA-like neurons are shown on coronal diagrams encompassing the relevant dopaminergic, midbrain subregions (Supplementary Figure 1a).

Data analysis. The spontaneous basal activity was recorded in each putative DA-like neuron for at least 100 s before the drug was applied. For further analysis, two 20 s-long periods were used, one of which referred to basal activity and one that was performed during NMDA application. The calculation of action potential durations and spontaneous firing rates, the analysis of interspike intervals, and the

detection and analysis of bursts of action potentials were performed off-line using custom-made Spike2 scripts (Cambridge Electronic Design Inc.; Cambridge, UK). Bursts were detected based on the criteria of Grace and Bunney[2]. An analysis of burst parameters was performed only in the cells that generated at least one burst within the subjected period. The pattern of activity in the recorded DA-like neurons was classified as either non-bursting or bursting based on the percentage of action potentials that were generated in bursts (<10%: non-bursting; >=10%: bursting).

Rotarod

On the first day, the mice were placed on the rod and allowed to habituate to the apparatus in three 5-minute trials during which the rotation was set to 6 rpm. Over the next three days, the animals were tested in three trials per day at 30 minute intervals. Every trial started with 5 minutes of adaptation, which was followed by 5 minutes of acceleration from 6 to 40 rpm. Time to fall was averaged from three trials for each animal.

References:

1. Paxinos, G. & Franklin, K. B. J. *The Mouse Brain in Stereotaxic Coordinates (Deluxe Edition), Second Edition*. (Academic Press, 2001).
2. Grace, A. A. & Bunney, B. S. The control of firing pattern in nigral dopamine neurons: burst firing. *J. Neurosci.* **4,** 2877–2890 (1984).

Supplementary figures

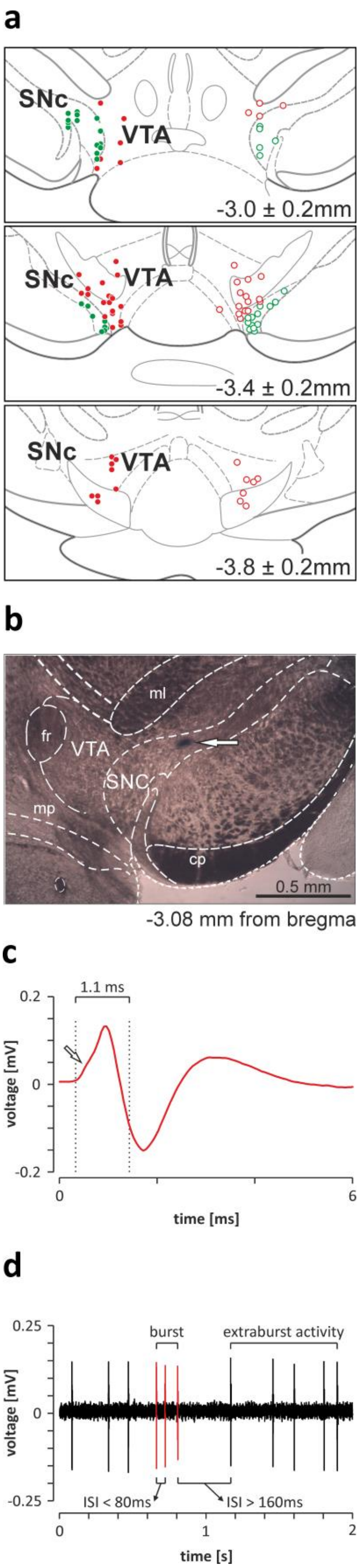

Figure S1. Histological verification of recording sites and electrophysiological criteria used to classify neurons as DA-like. (a) Localization of recorded cells in control animals (left side; ● - VTA, ● - SNc) and mutant animals (right side; ○ - VTA, ○ - SNc) depicted on coronal sections based on mouse brain stereotaxic atlas. Anterior-posterior distance from bregma point is indicated for each section. (b) Example of coronal section from mouse brain with recording site marked by Chicago Sky Blue deposition within SNc. (c) Typical, three phasic waveform of extracellularly recorded action potential (AP) of a DA-like neuron. Length of AP is measured from AP initiation to the trough of the negative phase. The dashed lines mark AP length criterion (>1.1ms) for identification of DA-like neurons. Arrow marks notch, usually visible in AP shape of DA-like neurons. (d) Spontaneously occurring burst (red spikes) recorded in DA-like neurons, consisting of at least 2 action potentials with decreasing amplitude and increasing duration of interspike interval. Criteria of burst detection are marked bellow the signal.

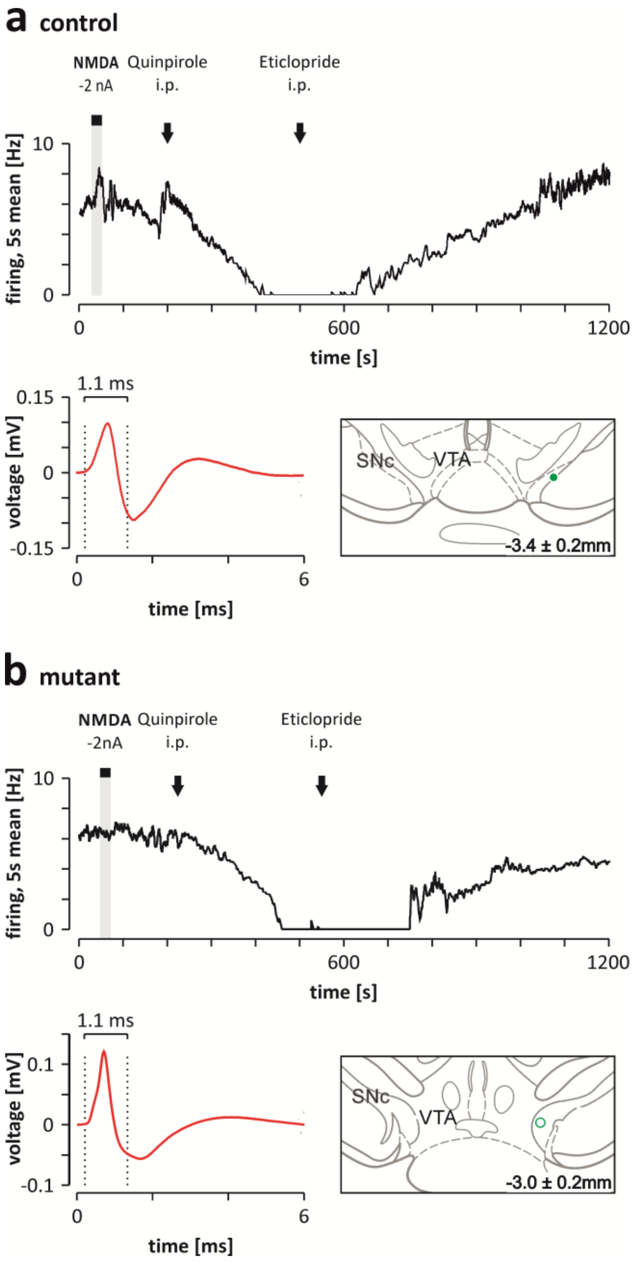

Figure S2. Pharmacological identification of DA-neurons with D2 receptor agonist (Quinpirole) and antagonist (Eticlopride). (a) Effect of i.p. injections of quinpirole and eticlopride on spontaneous activity of DA-like neuron in SNc of control animal. Top panel depicts firing rate of single DA-like neuron in baseline conditions, during NMDA iontophoretic application (marked by grey area) and after i.p. injection of quinpirole followed by eticlopride. Time of drugs' i.p. injections is marked with arrows. Bottom left panel shows averaged extracellular action potential (AP) waveform of tested DA-like neuron. The dashed lines mark AP length criterion for identification of DA-like neurons. Bottom right panel depicts location of recorded neuron (●) on coronal section from mouse brain atlas. (b) Effect of i.p. injections of quinpirole and eticlopride on spontaneous activity of DA-like neuron in SNc of mutant animal. Top panel depicts firing rate of single DA-like neuron in baseline conditions, during NMDA iontophoretic application (marked by grey area) and after i.p. injection of quinpirole followed by eticlopride. Time of drugs' i.p. injections is marked with arrows. Bottom left panel shows averaged extracellular action potential (AP) waveform of tested DA-like neuron. The dashed lines mark AP length criterion for identification of DA-like neurons. Bottom right panel depicts location of recorded neuron (○) on coronal section from mouse brain atlas.

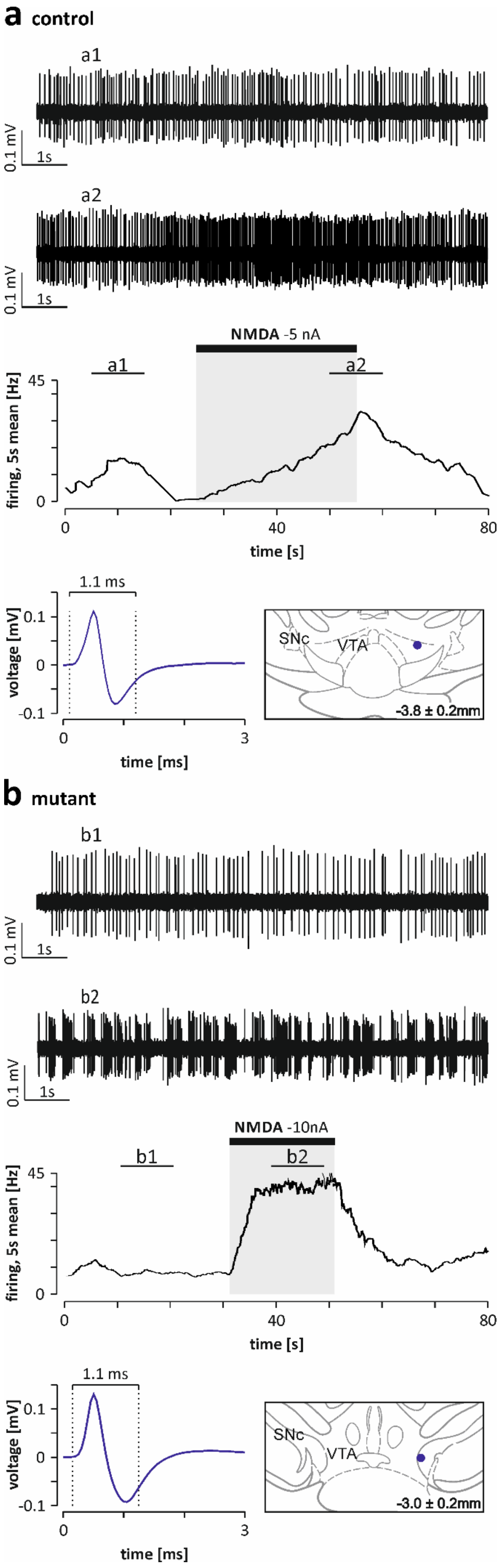

Figure S3. Response of non-DA neurons to iontophoretically applied NMDA. (a) An example of recorded non-DA neuron in VTA of control animal. Central panel depicts averaged firing rate of single non-DA neuron during baseline (raw signal on panel „a1”) and NMDA application (raw signal on panel „a2”). Time of drug application is marked by grey area. Bottom left panel represents averaged extracellular action potential (AP) waveform of non-DA neuron. The dashed lines mark AP length criterion for identification of DA-like neurons. Bottom right panel depicts location of recorded neuron (●) on coronal section from mouse brain atlas. (b) An example of recorded non-DA neuron in SNc of mutant animal. Central panel depicts averaged firing rate of single non-DA neuron during baseline (raw signal on panel „b1”) and NMDA application (raw signal on panel „b2”). Time of drug application is marked by grey area. Bottom left panel represents averaged extracellular action potential (AP) waveform of non-DA neuron. The dashed line marks AP length criterion for identification of DA-like neurons. Bottom right panel depicts location of recorded neuron (●) on coronal section from mouse brain atlas.

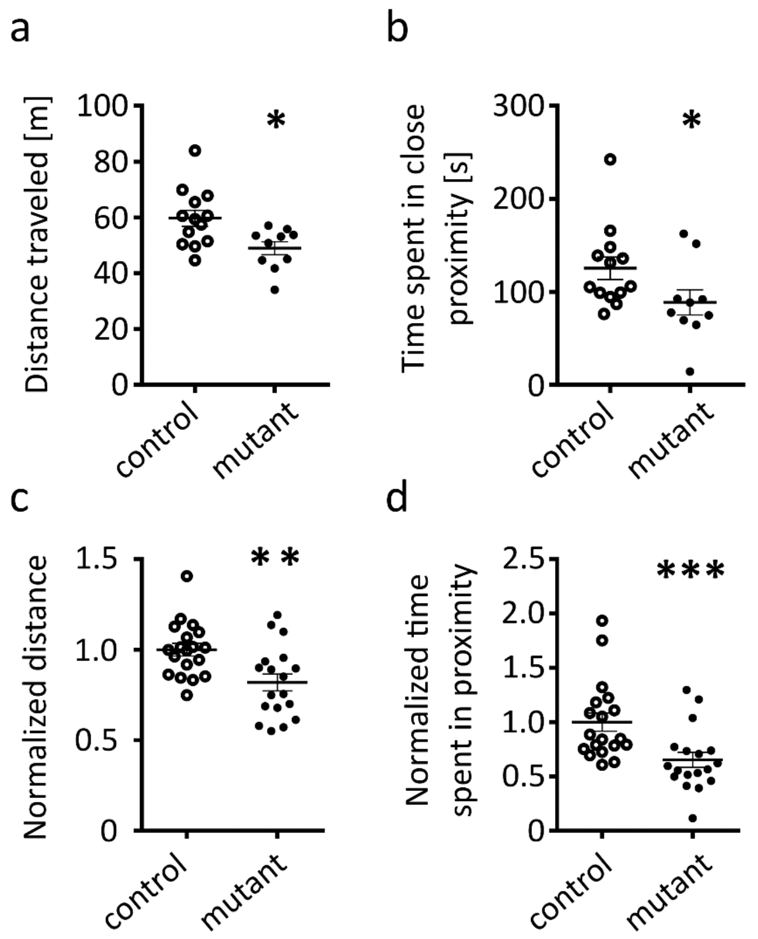

Figure S4. Replication cohort for the social interaction experiment. The graphs summarize data from an additional cohort of mice that was tested in the social interaction paradigm. (a) Distance travelled during the 10 minute interaction period and (b) time spent in close proximity (> 5 cm between body centers). (c,d) Pooled data from the two social interactions experiments, normalized to respective control means. Normalization was necessary due to a basal (genotype-independent) difference between cohorts. Error bars show the s.e.m., ‘*’ indicates Mann-Whitney $p < 0.05$, ‘**’ is $p < 0.01$ and ‘***’ indicates $p < 0.001$.

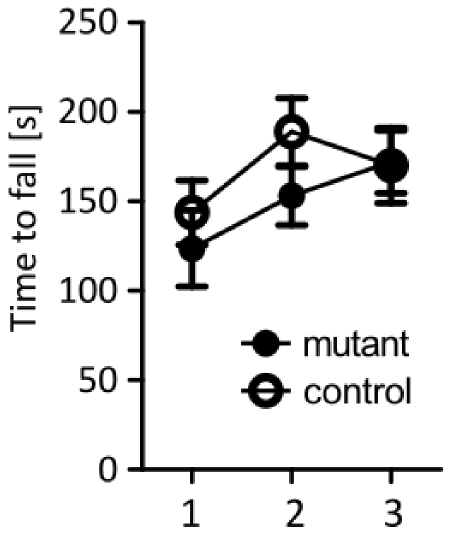

Figure S5. Motor skills measured in rotarod test. The graph shows the mean latency to fall from the rod calculated from three trials in mutant (n=8) and control (n=6) mice during consecutive three days.

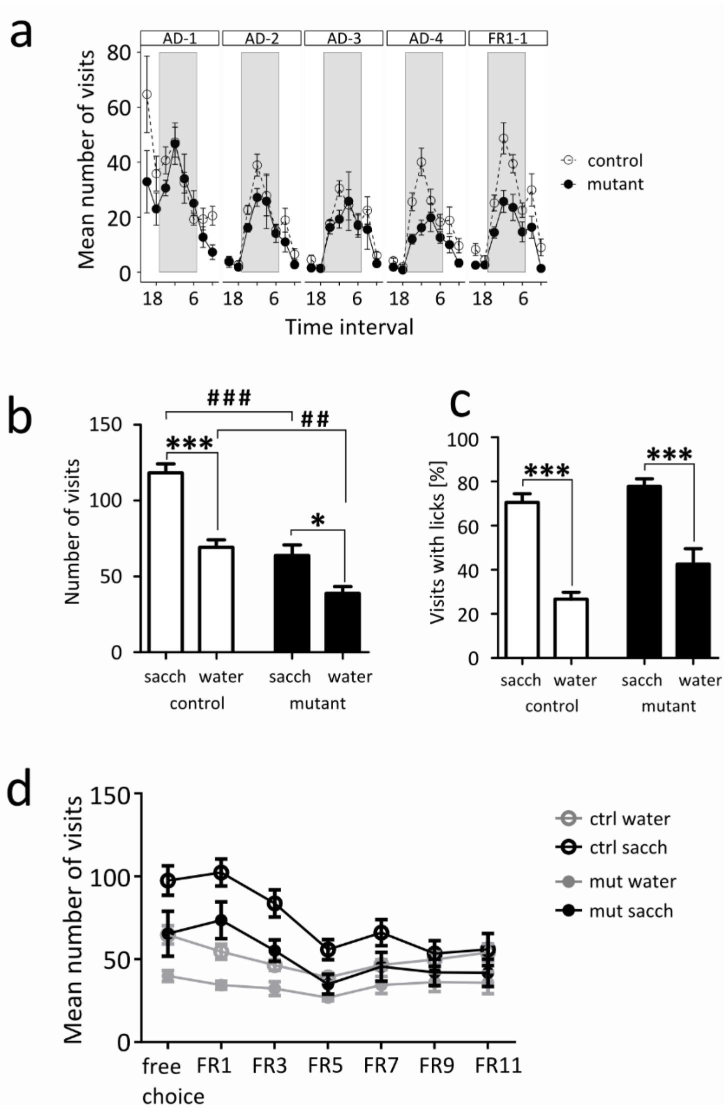

Figure S6. (a) Circadian activity. The graph shows the mean number of visits shown in 3 h bins that separate the 5 days of the experiment. (b) Mean number of visits to the saccharin and water corners by the mutant and control mice under a fixed ratio 1 (FR1) schedule. (c) The fraction of visits that included drinking. (d) Mean number of visits during the effort-based test in an IntelliCage.

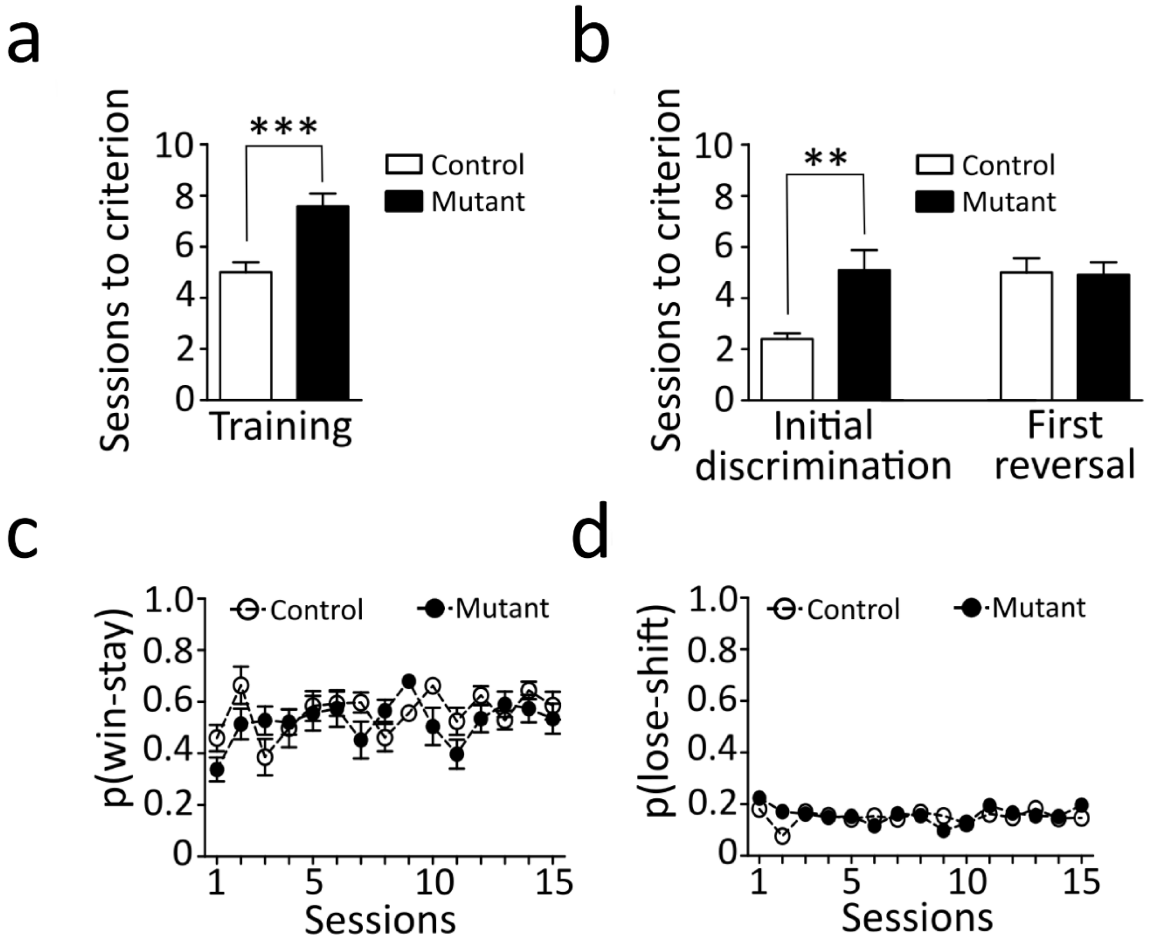

Figure S7. Sensitivity to positive and negative feedback. (a) The graph shows the mean number of sessions that were required to reach the criterion during the training phase of the probabilistic reversal learning (PRL) task. (b) The mean number of sessions that were required to reach the criterion during the initial discrimination phase and after the first reward contingency reversal. (c) The probability of selecting the same choice option as before after a rewarded trial (win-stay). (d) The probability of selecting an alternative choice option after a non-rewarded trial (lose-shift). A total of 13 mutant & 12 control animals started training (a) and 10 mutant & 11 control mice met the inclusion criteria for the main phase of the experiment (b-d). Error bars show the s.e.m., '*' indicates t-test $p < 0.01$, and '***' indicates $p < 0.001$.

Supplementary tables

Table S1. Spontaneous firing rate of DA-like neurons in VTA and SNc of control and mutant mice.

	VTA		SNc	
	control (n =34)	mutant (n = 26)	control (n =22)	mutant (n = 18)
**total firing rate [Hz]**	6.0 ± 0.4	4.0 ± 0.3 ***[1]	6.6 ± 0.5	4.0 ± 0.4 ***[1]
**extraburst firing rate [Hz]**	4.8 ± 0.4	3.8 ± 0.4	5.7 ± 0.5	3.8 ± 0.3 **[1]

[1] - Unpaired Student's t-test

Table S2. Firing rate of DA-like neurons in VTA and SNc of control and mutant mice under baseline conditions and during NMDA application.

	VTA			SNc		
		**baseline**	**NMDA**		**baseline**	**NMDA**
**total firing rate [Hz]**	control (n = 24)	5.7 ± 0.4	10.0 ± 0.5	control (n = 19)	6.5 ± 0.5	10.8 ± 0.7
	mutant (n = 25)	4.0 ± 0.4	4.1 ± 0.4	mutant (n = 16)	4.1 ± 0.4	4.1 ± 0.4
**extraburst firing rate [Hz]**	control (n = 14)	4.6 ± 0.5	5.6 ± 0.8	control (n = 11)	5.7 ± 0.6	7.7 ± 1.1
	mutant (n = 10)	3.8 ± 0.4	3.9 ± 0.4	mutant (n = 7)	3.9 ± 0.3	3.9 ± 0.4

Table S3. Two-way ANOVA analysis of firing rate of DA-like neurons in VTA and SNc of control and mutant mice under baseline conditions and during NMDA application.

	VTA			SNc		
	**genotype**	**NMDA** (treatment)	**genotype x NMDA** (interaction)	**genotype**	**NMDA** (treatment)	**genotype x NMDA** (interaction)
**total firing rate [Hz]**	$F_{1,47} = 51.98$ $p<0.0001$****	$F_{1,47} = 164.3$ $p<0.0001$****	$F_{1,47} = 142.2$ $p<0.0001$****	$F_{1,33} = 40.97$ $p<0.0001$****	$F_{1,33} = 79.18$ $p<0.0001$ ****	$F_{1,33} = 79.14$ $p<0.0001$****
**extraburst firing rate [Hz]**	$F_{1,47} = 3.46$ $p=0.07$	$F_{1,47} = 4.631$ $p=0.04$*	$F_{1,47} = 3.097$ $p=0.085$	$F_{1,33} = 9.404$ $p=0.004$**	$F_{1,33} = 7.414$ $p=0.01$**	$F_{1,33} = 6.373$ $p=0.02$*

Table S4. Parameters describing spontaneous (baseline) and NMDA induced bursting of DA-like neurons in control and mutant mice.

	VTA			SNc		
		**baseline**	**NMDA**		**baseline**	**NMDA**
**%SFB [%]**	control (n = 24)	18 ± 6	48 ± 8	control (n = 19)	15 ± 5	39 ± 9
	mutant (n = 25)	7 ± 3	8 ± 4	mutant (n = 16)	6 ± 2	4 ± 2
**bursting rate [Hz]**	control (n = 14)	0.5 ± 0.2	1.2 ± 0.2	control (n = 11)	0.5 ± 0.2	1.0 ± 0.2
	mutant (n = 10)	0.2 ± 0.1	0.2 ± 0.1	mutant (n = 7)	0.2 ± 0.1	0.2 ± 0.1
**spikes per burst**	control (n = 14)	2.8 ± 0.3	5.9 ± 0.8	control (n = 11)	2.7 ± 0.4	6.4 ± 0.7
	mutant (n = 10)	2.2 ± 0.1	2.8 ± 0.2	mutant (n = 7)	2.7 ± 0.3	2.3 ± 0.3
**intraburst ISI [ms]**	control (n = 14)	38.0 ± 4.5	38.7 ± 2.2	control (n = 11)	44.4 ± 5.0	45.6 ± 2.7
	mutant (n = 10)	33.5 ± 2.9	39.9 ± 5.5	mutant (n = 7)	42.1 ± 4.8	33.2 ± 5.6
**burst length [ms]**	control (n = 14)	116.9 ± 22.2	225.6 ± 33.4	control (n = 11)	127.5 ± 28.6	283.5 ± 32.7
	mutant (n = 10)	73.4 ± 7.4	111.6 ± 20	mutant (n = 7)	118.8 ± 25.5	80.8 ± 24.3

Table S5. Two-way ANOVA analysis of parameters describing spontaneous (baseline) and NMDA induced bursting of DA-like neurons in control and mutant mice.

	VTA			SNc		
	**genotype**	**NMDA** (treatment)	**genotype x NMDA** (interaction)	**genotype**	**NMDA** (treatment)	**genotype x NMDA** (interaction)
**%SFB [%]**	$F_{1,47}$ = 13.22 p=0.0007***	$F_{1,47}$ = 13.22 p=0.0007***	$F_{1,47}$=20.01 p<0.0001****	$F_{1,33}$ = 9.15 p = 0.005 **	$F_{1,33}$ = 11.05 p = 0.002 **	$F_{1,33}$ = 15.19 p = 0.0004 ***
**bursting rate [Hz]**	$F_{1,22}$ = 16.20, p=0.0006 ***	$F_{1,22}$=13.70, p=0.001 ***	$F_{1,22}$=12.44, p=0.002 **	$F_{1,16}$=10.70, p=0.005 **	$F_{1,16}$=6.51, p=0.02 *	$F_{1,16}$=7.40, p=0.02 *
**spikes per burst**	$F_{1,22}$ = 9.30 p=0.006**	$F_{1,22}$ = 16.12 p=0.0006***	$F_{1,22}$ = 7.30 p=0.01**	$F_{1,16}$ = 12.88 p=0.003**	$F_{1,16}$ = 11.31 p=0.004**	$F_{1,16}$ = 18.17 p=0.0006***
**intraburst ISI [ms]**	$F_{1,22}$ = 0.15 p=0.7	$F_{1,22}$ = 0.95 p=0.3	$F_{1,22}$ = 0.60 p=0.5	$F_{1,16}$ = 1.56 p=0.2	$F_{1,16}$ = 1.77 p=0.2	$F_{1,16}$ = 3.12 p=0.1
**burst length [ms]**	$F_{1,22}$ = 8.99 p=0.007**	$F_{1,22}$ = 8.64 p=0.008**	$F_{1,22}$ = 1.99 p=0.2	$F_{1,16}$ = 7.53 p=0.02*	$F_{1,16}$ = 8.42 p=0.01**	$F_{1,16}$ = 22.79 p=0.0002***

Table S6. Statistical analysis of behavior in the probabilistic reversal test.

	Control (N = 10)	NR1DATCreERT2 (N = 11)	p value (t test)
**Between-session**			
Trials per session	89.62 ± 4.44	76.88 ± 6.37	0.12
Reversals	1.7 ± 0.15	1.64 ± 0.24	0.83
Trial time	28.71 ± 1.29	34.23 ± 3.06	0.12
Response latency	16.06 ± 1.29	21.97 ± 2.9	0.09
Reward latency	2.69 ± 0.43	2.46 ± 0.32	0.67

	Control (N = 13)	NR1DATCreERT2 (N = 12)	p value (t test)
**Within-session**			
Trials per session	129.8 ± 8.63	128.8 ± 10.16	0.94
Reversals	3.12 ± 0.41	3.92 ± 0.51	0.23
Trial time	29.34 ± 2.1	31.01 ± 3.04	0.65
Response latency	15.01 ± 1.51	18.06 ± 2.62	0.31
Reward latency	2.87 ± 0.68	1.71 ± 0.11	0.12
